# Increased radical scavenging activity of thymoquinone and l-ascorbic acid dual encapsulated in palmitoyl-chitosan nanoparticles in a human normal lung fibroblast, MRC-5 due to synergistic antioxidative effects

**DOI:** 10.1039/d3ra04326f

**Published:** 2023-09-20

**Authors:** Nurhanisah Othman, Siti Nurul Ain Md. Jamil, Mas Jaffri Masarudin, Ruqayyah Ainul Bashirah Mohd Jusoh, Mohammed Numan Alamassi

**Affiliations:** a Chemistry Department, Faculty of Science, Universiti Putra Malaysia 43400 UPM Serdang Malaysia ctnurulain@upm.edu.my; b Centre of Foundation Studies for Agricultural Science, Universiti Putra Malaysia 43400 UPM Serdang Malaysia; c Department of Cell and Molecular Biology, Faculty of Biotechnology and Biomolecular Sciences, Universiti Putra Malaysia Serdang 43400 Selangor Malaysia; d UPM-MAKNA Cancer Research Laboratory, Institute of Biosciences, Universiti Putra Malaysia Serdang 43400 Selangor Malaysia

## Abstract

Less effective antioxidant supplementation in combating free radicals is often related to the lack of the formulation of carriers. The antioxidant may be one of the most powerful substances but is marred by poor uptake by cells when the carrier degraded and dissolved too rapidly. Nanoparticle (NP) systems are promising in overcoming the problem since they provide high surface area to enhance encapsulation and release efficiency. With the right selection of material, NP carriers could function as constructive antioxidant cargos. Generally, NPs carry only one active ingredient; this study, however, utilized chitosan nanoparticles (CNPs) and hydrophobically modified palmitoyl-chitosan nanoparticles (PCNPs) that were dual encapsulated with antioxidants of different polarities, namely, hydrophobic thymoquinone (TQ) and hydrophilic l-ascorbic acid (LAA) to evaluate their combination effects in scavenging free radicals. The antioxidants followed zero-order release kinetics with a controlled release manner for about 48 h. The interaction effects between TQ and LAA loaded in the NP systems were determined by classical isobologram (CI) values. The CI values were derived by a diphenyl picrylhydrazyl (DPPH) assay, a radical scavenging activity assay. Combined TQ and LAA had CI values of less than one, with a lower value in the PCNP system than in the CNP system. This indicates that the interaction between those antioxidants showed higher synergistic effects in PCNPs, which enhanced the DPPH radical scavenging activities. The antioxidative potential of compound(s) encapsulated in the PCNP carrier was further experimented by a reactive oxygen species (ROS) assay on a human normal lung fibroblast cell line (MRC-5) as lung is one of the organs with high accumulation of free radicals. About 48 h post treatment, the dual-loaded TQ and LAA in PCNPs showed the lowest ROS level in comparison to single-loaded antioxidants and bare antioxidant delivery. The hydrogen peroxide (H_2_O_2_) radical scavenging was influenced by both the controlled release property of the PCNP system and the synergy between TQ and LAA. In short, dual-loaded TQ and LAA in the hydrophobically modified PCNP had effectively depicted the capability of a single CS-based nanocarrier to hold more than one compound at a time to function as a potent radical scavenger.

## Abstract 2

Less effective antioxidant supplementation in combating free radicals is often related to the lack of the formulation of carriers.

## Graphical abstract

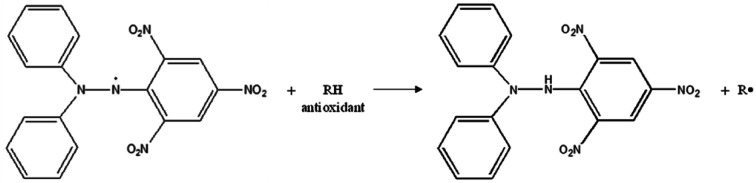

## Introduction

Free radicals are atoms, molecules or ions with unpaired electrons, which are naturally produced in the human body *via* metabolic processes such as food conversion to energy and during exercise. Reactive oxygen species (ROS) is the most common free radical in living tissues that consists of oxygen; superoxide anions, singlet oxygen, lipid peroxides and hydroxyls are few examples of ROS.^1^ Although free radicals are essential for apoptosis, ion transportation, gene expression and cell signaling, uncontrolled accumulation of free radicals might lead to oxidative stress and later cause chronic diseases such as atherosclerosis, arthritis, cancers, cardiovascular diseases, diabetes mellitus, neurological disorders and premature aging *via* deoxyribonucleic acid (DNA), protein and lipid alteration. As they are unstable and highly reactive, they tend to capture an electron from another substance, causing that substance to lose an electron; thus, promoting a chain reaction process of oxidation.^2,3^

In order to even out the free radicals, external sources of antioxidants are essential on top of the limited human naturally produced antioxidants, gluthathione (GSH) and antioxidant enzyme, superoxide dismutase (SOD). HydrophobicTQ, an active ingredient in *Nigella sativa* or known as black seed, has excellent antioxidant, anticancer and anti-inflammatory properties.^4^ Studies reported its potent antioxidative function in attenuating neurotoxic effects and oxidative stress in the nervous system, as an impact of exposure to arsenic.^5^ Furthermore, hydrophilic LAA is a powerful antioxidant that can assist in reducing cell damage by scavenging free radicals due to the presence of an enediol moiety.^6^ Furthermore, TQ and LAA were combined and successfully attenuated bisphenol-A, BPA (a chemical used to produce polycarbonate), induced hepatorenal toxicity and oxidative stress in adult male albino rats as a result of the synergistic effects of TQ and LAA. TQ had a high ability in scavenging radicals but the performance is marred by poor bioavailability. Therefore, a golden standard of antioxidant, LAA, was determined to be the most effective assistance to counter the matter. In the same study, it was found that the level of naturally produced antioxidants, GSH and SOD, in the liver of BPA-induced rats treated with both TQ and LAA increased. This specifies that the combination of TQ and LAA produced synergistic interaction and elevated the antioxidant activity in the liver.^7^ For the purpose of increasing the efficiency of radical scavenging by combined TQ and LAA, they were encapsulated in a NP. The nanosized particle exhibits a large surface area, which increases the efficiency of encapsulation and release of compounds.^9,10^ The utilization of NPs to contain TQ and LAA had been reported to increase the bioavailability and cellular uptake due to the controlled release over 48 h.^8^ Chitosan (CS)-based NPs were used in this study, as they have many versatile hydroxyl and amino groups that are useful for modifications.^11^ Since CS is hydrophilic and one of the compounds encapsulated is hydrophobic, which is TQ, the CNP was slightly modified with palmitic acid to be partially hydrophobic. The attachment of a palmitoyl group to the amine of CS was described to significantly increase the encapsulation and release of TQ as well as LAA.^8^ In our earlier research, the synthesis, optimization and encapsulation of TQ and LAA in two different systems, CNPs and PCNPs were reported and further employed for antioxidative capacity evaluation.^8,12^

**Fig. 1:**
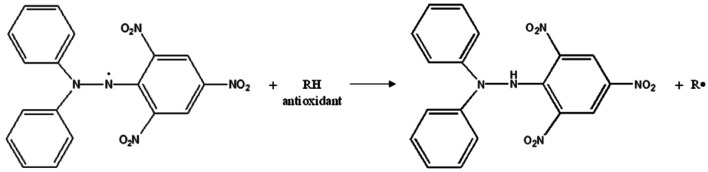
Reduction of DPPH by an antioxidant *via* donation of a hydrogen atom.

In this study, the antioxidant activities of TQ and LAA encapsulated in CNP and PCNP systems against free radicals were determined using two assays. First is the 1,1-diphenyl-2-picrylhydrazyl (DPPH) assay, a stable nitrogen free radical that can be measured by colorimetric analysis at a specific wavelength, 517 nm, using a microplate reader.^13^ Many studies have been reporting the effectiveness of using DPPH as a measure of antioxidative properties, despite its easy handling.^3,14,15^ DPPH considers both antioxidant concentration and scavenging reaction time to reach a plateau.^16^ DPPH can only dissolve in organic solvents and its absorbance in methanol and acetone decreases when exposed to light. The presence of DPPH radicals was highly detected in its purple solution, but this declined as the color faded and turned yellow, indicating free radicals' reduction due to antioxidative action. The discoloration of DPPH solution is influenced by the existence of compounds capable of either donating hydrogen or transferring an electron.^17,18^ An example of hydrogen transfer process from an antioxidant to the DPPH radical is shown in Fig. 1.^15,19^

The aim to use TQ and LAA for DPPH scavenging is fulfilled by verifying whether dual encapsulation of these two potent antioxidants could increase the efficacy in reducing free radicals, or not. This could be determined by finding the EC_50_ value of each compound in all state of systems; bare, single loaded or dual loaded. The EC_50_ value is the concentration of a compound that gives half-maximal response. By comparing the EC_50_ values, with the aid of CI, in a study of drug interactions based on dose and effect factors, the effect of TQ and LAA combination could be determined, which could be either additive, synergistic or antagonistic.^20^ A combination is synergistic when the combined effect is larger than the additive effect of each drug. Likewise, a combination is antagonistic when the combined effect is smaller than the additive effect of each drug.^21^ By knowing that, it can then be concluded whether the combination improves the scavenging efficiency or not.

**Fig. 2:**
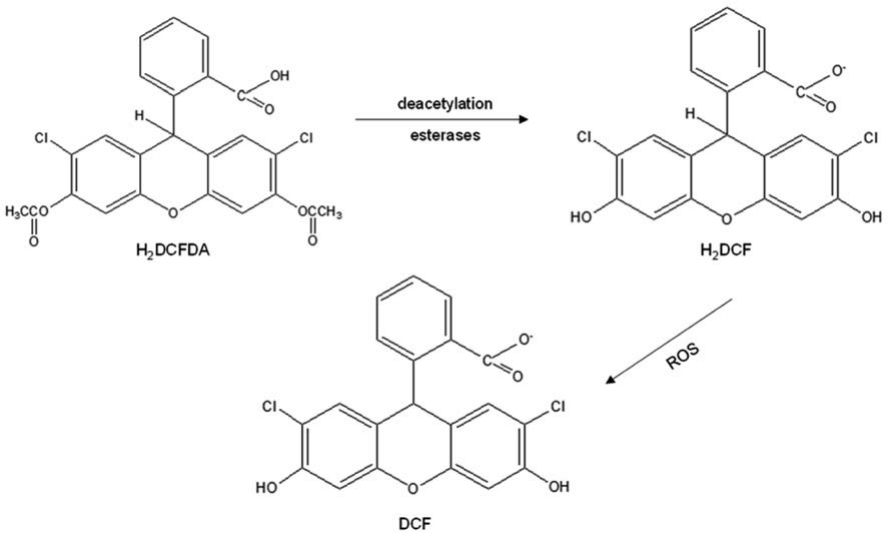
Conversion of DCFDA into fluorescent DCF (dichlorofluorescein) that resembles the presence of ROS.

The reactive oxygen species (ROS) assay is another option to quantify ROS produced by using a cell-permeable 5-(and-6)-chloromethyl-2′,7′-dichlorodihydrofluorescein diacetate, acetyl ester (CM-H_2_DCFDA) probe. Once the probe diffuses into a cell, esterases deacetylate H_2_DFCDA into dicholorodihydrofluorescein (H_2_DCF) at two esterase bonds. Next, oxidation occurs in the presence of ROS such as hydroxyl radical, hydroperoxides, and peroxynitrite, but most effectively H_2_O_2_, and oxidizes H_2_DCF to fluorescent dichlorofluorescein (DCF) that can be measured by flow cytometry at excitation and emission wavelengths of 488 and 525 nm, respectively.^22,23^ The conversion of DCFDA into DCF is shown in Fig. 2. In a study, it was reported that particulate matter of less than 10 μm, PM10, was introduced in a normal human lung fibroblast cell line, MRC-5, which resulted in inflammation and increased ROS production. Transient receptor potential melastatin 2 (TRPM2) blockers, clotrimazole (CLZ) and anthranilic acid (ACA) were then used to attenuate H_2_O_2_ produced by the cells. The efficacious reduction of H_2_O_2_ levels was seen by lesser fluorescence DCF appearance, following CLZ and ACA treatment. This concludes that the ROS assay is a reliable test for the detection of ROS presence in cells.^22^ A high ROS level was observed in a lung cancer patient, which was mostly contributed by cigarette smoke that contains various carcinogens.^24^ In addition, human coronavirus 229E (HCoV-229E) infection elevated ROS production due to the suppression of NRF-2, a regulator of cellular resistance to oxidants.^25^ Hence, in this study, a normal human lung fibroblast cell line, MRC-5, was used as target cells for the ROS assay test since lung is a site for ROS accumulation.

Based on the reported convincing outcomes of the combination of TQ and LAA in chitosan-based NPs (CNPs and PCNPs), this study intended to discover the antioxidative potentials in scavenging free radicals by DPPH and ROS assays. Both systems, CNPs and PCNPs, were compared to determine which of the systems has the most effective scavenging activity. According to reported data, the PCNP had elevated encapsulation and release efficiency due to the conjugation of a palmitoyl group, as compared to the CNP.^8,12^ This could be an indicator of what has to be expected in this study. After all, with good interactions between these antioxidants as well as the right polymer for the carrier, promising antioxidative NPs are close to reality.

## Materials and method

### Materials

A chitosan (CS) with MW = 50 000–190 000 Da was used as a carrier, and tripolyphosphate (TPP) with MW = 367.86 Da was used as a crosslinking agent. Both were purchased from Sigma-Aldrich (St. Louis, MO, USA). Glacial acetic acid, sodium hydroxide (NaOH) pellets, 5% w/v hydrochloric acid (HCl), l-ascorbic acid (LAA), dimethylsulfoxide (DMSO) and 30% hydrogen peroxide (H_2_O_2_) were purchased from R&M Chemicals (United Kingdom). Thymoquinone (TQ) and palmitic acid *N*-hydroxysuccinimide (NHS) ester were purchased from Sigma Aldrich (St. Louis, MO, USA). Absolute ethanol was purchased from Systerm (Shah Alam, Selangor, Malaysia). Phosphate buffered saline (PBS) pellets were purchased from MP Biomedicals (Solon, OH, USA). 1,1-Diphenyl-2-picrylhydrazine (DPPH) was purchased from Sigma Aldrich (St. Louis, MO, USA). Gallic acid was purchased from Merck (Darmstadt, Germany). All chemicals were of analytical grade and used without further purification. Reactive oxygen species (ROS) assay probe, 5-(and-6)-chloromethyl-2′,7′-dichlorodihydrofluorescein diacetate, CM-H_2_DCFDA, was purchased from Invitrogen by Thermo Fisher Scientific (Waltham, MA, USA). Normal human lung fibroblast cell line (MRC-5) was provided by the American Tissue Culture Collection (ATCC) (Manassas, VA, USA). Roswell Park Memorial Institute-1640 (RPMI-1640) media and 0.4% trypan blue solution were purchased from Gibco by Life Technologies (Carlsbad, CA, USA). Fetal bovine serum (FBS) was purchased from Biosera (Nuaille, France). Antibiotic-antimycotic mixed stock (100×) and 2.5 g L^−1^-Trypsin/1 mmol L^−1^-EDTA solution (1×) were purchased from Nacalai Tesque (Tokyo, Japan).

### Preparation of nanoparticle samples

#### Synthesis of chitosan nanoparticles, CNPs

Chitosan nanoparticles (CNPs) were prepared by an ionic gelation method, which involves the crosslinking reaction of the positively charged CS and negatively charged TPP.^26–31^ First, ∼1.0 mL of 1.0% acetic acid was added to the low-molecular weight CS to dissolve it and then further diluted to form a working solution concentration of 0.5 mg mL^−1^ CS. Next, the pH of the CS was adjusted to 5.0 by adding a 1 M aqueous sodium hydroxide solution. The crosslinker TPP was dissolved in distilled water at a concentration of 0.7 mg mL^−1^, the pH of which was then altered to 2.0 with 1.0 M hydrochloric acid. Primarily, the CNP was formed by adding 600 μL of CS to a range volume of TPP solution (0 to 300 μL). The optimum volume of TPP solution that met the targeted size and other characteristics is explained in the results and discussion part. The mixture was then centrifuged at 13 000 rpm for 20 min for purification.

#### Encapsulation of thymoquinone and l-ascorbic acid into chitosan nanoparticles, CNP-TQ-LAA

A single TQ-loaded CNP known as CNP-TQ was prepared by dissolving approximately 2.0 mg of TQ in 2.0 mL of 99.0% of DMSO, making a stock concentration of 6.1 mM. It was further diluted to three different intermediate concentrations. Only 100 μL of each diluted TQ was added to three different tubes of 600 μL of 0.5 mg mL^−1^ of CS. Finally, 250 μL of 0.7 mg mL^−1^ of TPP was added to produce CNP-TQ. The final TQ concentrations used were 100, 150 and 200 μM.

Another single LAA-loaded CNP, named CNP-LAA, was prepared by first pouring approximately 10.0 mg of LAA into 10.0 mL of 0.7 mg mL^−1^ TPP solution, making a LAA stock concentration of 5.7 mM. It was further diluted to three different intermediate concentrations. Then, 250 μL of each diluted LAA was added into three different tubes of 600 μL of 0.5 mg mL^−1^ CS solutions, to produce CNP-LAA. The final LAA concentrations used were 160, 235 and 310 μM.

**Fig. 3:**
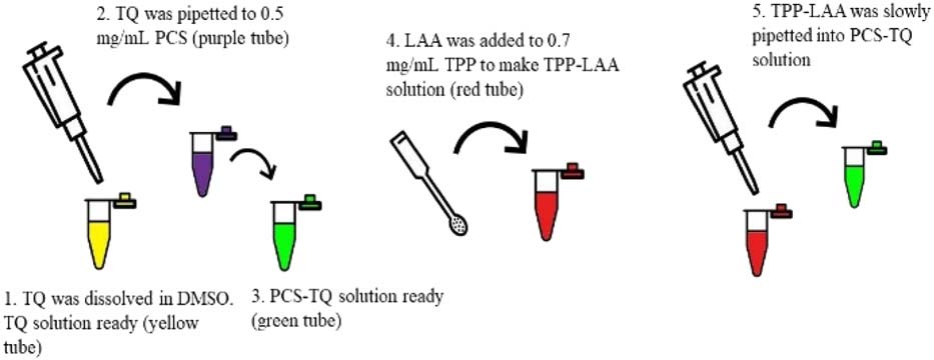
Methodology of dual encapsulation of TQ and LAA into PCNPs.

The encapsulation of both compounds, TQ and LAA, to make CNP-TQ-LAA is shown in Fig. 3. The TQ and LAA stock solutions were prepared as described above. A set of TQ concentrations (100, 150 and 200 μM) with a set of LAA concentrations (160, 235 and 310 μM) were tested to find the optimum formulation (with the best particle size, dispersion and encapsulation efficiency). About 100 μL of diluted TQ solution was added to 600 μL of 0.5 mg mL^−1^ of CS, and the solution was mixed for a while. Then, 250 μL of LAA diluted solution was poured into the mixture of TQ and CS.

#### Synthesis of palmitic acid modified chitosan nanoparticles, PCNP

For the purpose of modifying CS to be more hydrophobic, palmitic acid NHS ester was added in the synthesis process. CS powder was dissolved in 1.0% acetic acid and distilled water to form a concentration of 1.0 mg mL^−1^ CS master solution (CS MS). Separately, palmitic acid NHS ester was dissolved in absolute ethanol to a concentration of 0.36 mg mL^−1^. Then, the CS solution was adjusted to pH 6. A palmitic acid NHS ester solution was added into the CS solution dropwise, with a volume ratio of 1 : 2, and the mixture was left at 50 °C for 20 h to react. After the incubation was completed, the solution was adjusted to pH 9 using 1 M NaOH. Then, it was centrifuged at 2200×*g* for 45 min to form a precipitate. Following precipitation, it was washed once with 50 : 50 acetone: absolute ethanol and twice with distilled water. The supernatant was removed from each centrifugation cycle. The precipitate was dried in an oven at 50 °C for 72 h. This process produced pellet, which is called palmitoyl-chitosan (PCS).^32^ The PCS pellet will be used to synthesize PCNP, LAA-encapsulated PCNP (PCNP-LAA), TQ-encapsulated PCNP (PCNP-TQ) and PCNP-LAA-TQ.

The PCS pellet was dissolved with 1.0% acetic acid and distilled water to a concentration of 1.0 mg mL^−1^. It was diluted to two-fold to get 0.5 mg mL^−1^ PCS working solution. Then, the solution was adjusted to pH 5 by adding 1 M aqueous NaOH solution. The crosslinker, TPP, was dissolved in distilled water to a concentration of 0.7 mg mL^−1^ and the pH was altered to 2.0 by using 1.0 M HCl. PCNP was formed by adding 600 μL of PCS to 250 μL of TPP. Then, the mixture was centrifuged at 13 000 rpm for 20 min to get purified PCNPs (this centrifugation step is only for unencapsulated PCNPs). Previously, it was found that 250 μL of TPP was an optimum volume to synthesize CNPs, and therefore, the same volume was used in this study to synthesize modified PCNPs. That optimum TPP volume was mainly determined based on the smallest empty carrier produced.^8,12^ This is because the expansion after encapsulation will be most minimal, which will enhance *in vivo* biological delivery.

#### Encapsulation of thymoquinone and l-ascorbic acid into palmitic acid-modified chitosan nanoparticles, PCNP-TQ-LAA

For a single encapsulation process, TQ was prepared by dissolving approximately 2.0 mg of TQ in 2.0 mL of 99.0% DMSO, making a stock concentration of 6.1 mM. Only 100 μL of diluted TQ was added into 600 μL of 0.5 mg mL^−1^ of PCS. Lastly, 250 μL of 0.7 mg mL^−1^ of TPP was dropped into the PCS-TQ mixture to produce PCNP-TQ. The final TQ concentration used was 150 μM.

Another single-loaded PCNP, PCNP-LAA, was prepared by first pouring approximately 10.0 mg of LAA into 10.0 mL of 0.7 mg mL^−1^ TPP solution, making an LAA stock concentration of 5.7 mM. Then, 250 μL of diluted LAA was added into 600 μL of 0.5 mg mL^−1^ PCS solution, to produce PCNP-LAA. The final LAA concentration used was 160 μM.

The encapsulation of both compounds, TQ and LAA, to make PCNP-TQ-LAA is shown in Fig. 3. Basically, the TQ and LAA solutions were prepared as mentioned previously. PCNP-TQ-LAA was prepared by mixing 100 μL of diluted TQ solution with 600 μL of 0.5 mg mL^−1^ of PCS for a while. Then, 250 μL of LAA-diluted solution was added into the mixture of TQ and PCS.^8^

### Encapsulation efficiency of thymoquinone and l-ascorbic acid into nanoparticles

The encapsulation efficiency (EE) was calculated by comparing the difference in absorbance of a total compound and free compound. Total compound refers to a compound solution only, while free compound refers to the compound that is unencapsulated in CNP-TQ, CNP-LAA, CNP-TQ-LAA, PCNP-TQ, PCNP-LAA or PCNP-TQ-LAA. Both solutions contained the same concentration of the compound. The % EE indicates the percentage of compound successfully encapsulated in the carrier; it was calculated using the following formula:^33,34^1
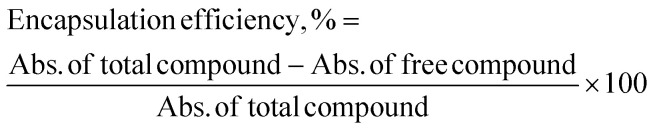


The absorbance was measured using a Lambda 35 UV-vis spectrophotometer (PerkinElmer, Waltham, MA, USA) at wavelengths of 257 nm and 267 nm for LAA and TQ, respectively. The wavelengths were determined based on the highest peak detected during the scanning of compounds. Triplicate test (*N* = 3) analysis of single and dual compounds loaded in CNPs and PCNPs was tested. All the stated procedures from section “Preparation of nanoparticles samples” to “Encapsulation efficiency of thymoquinone and l-ascorbic acid into nanoparticles” have been published in Othman 2020 and Othman 2018.^8,12^

### *In vitro* release of thymoquinone and l-ascorbic acid from CNPs and PCNPs

The encapsulated TQ and/or LAA in the CNP and PCNP carriers were freeze-dried using a Coolsafe 95-15 PRO freeze drier (ScanVac, Lynge, Denmark) for 48 h to remove water. The pellet obtained was then loaded into a quartz cuvette containing phosphate-buffered saline (PBS) pH 7.4 to mimic the physiological condition of human bloodstream.^35^ The sample was characterized using a UV-vis spectrophotometer (PerkinElmer, Waltham, MA, USA) that was set to wavelengths of 267 nm and 257 nm for TQ and LAA, respectively. It was left in the UV-vis spectrophotometer for 48 h, with auto data recording set for every 1 min (time drive option). A calibration curve for each compound was prepared as a reference to convert the absorbance reading to the concentration of the compound released. The percentage of compound released (%) was measured using the following formula, with the dilution factor taken into account:2
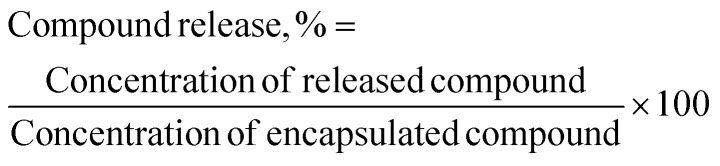


### Reactivity of diphenylpicrylhydrazyl (DPPH) radical towards thymoquinone and/or l-ascorbic acid encapsulated in CNPs and PCNPs by the DPPH assay

Antioxidant ability to scavenge radicals was determined by the DPPH assay adapted from Blois (1958).^36^ Eight samples CNP, CNP-LAA, CNP-TQ, CNP-LAA-TQ, PCNP, PCNP-LAA, PCNP-TQ and PCNP-LAA-TQ were tested using this assay. In a microcentrifuge tube, two-fold dilution of each sample was made in distilled water. 1,1-Diphenyl-2-picrylhydrazine DPPH powder was dissolved in DMSO and the working solution was prepared to 0.4 mM. In a 96-well plate, about 100 μL of 0.4 mM DPPH was added into each 100 μL sample. Separately, 100 μL of gallic acid standard solution and 100 μL of negative control using a DPPH solution were prepared. The plate was wrapped with an aluminium foil and incubated at room temperature for 0.5 h, 24 h and 48 h. The samples were read using a microplate reader (Bio-Tek Instrument, USA) at 517 nm. The assay and analysis were done in triplicate (*N* = 3), with three different synthesis batches. The radical scavenging activity of the antioxidants was calculated using the following formula:3
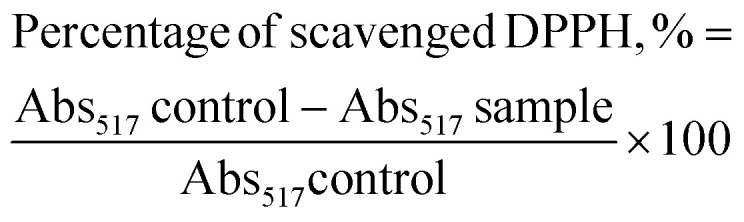
where Abs_517_ refers to the absorbances shown by the microplate reader at 517 nm wavelength.

Then, a linear line (*y* = *mx* + *c*) from a graph of percentage of scavenged DPPH *vs.* encapsulated compound concentration was plotted to determine the EC_50_ value of encapsulated compound, *x* when the *y* value is set to 50. The EC_50_ at this point refers to the concentration of encapsulated compounds required to scavenge 50% of the DPPH radicals. Since not all the encapsulated compounds were released, the percentage of released compounds (from release study data) must be taken into account to get the actual EC_50_ or known as EC_50_ of released compounds. The calculation for the EC_50_ value is as follows:

4 EC50 of released compound, μM = EC_50_ of encapsulated compound × Percentage of released compound

### Classical isobologram (CI) analysis to determine the synergy of thymoquinone and l-ascorbic acid release from CNPs and PCNPs

The combination of two therapeutic agents may not always result in greater efficiency than the sum of agents administered independently. In combinational therapies, the effects can be categorized into three types: additive, antagonistic and synergistic.^21^ CompuSyn, a computer software developed by Chou and Martin in 2005, can be used to determine the effect of combined therapeutics, represented as combination index (CI)-isobologram value. The quantitative CI value of <1, =1 and >1 indicates synergistic, additive, and antagonistic effects, respectively.^20,37^ For instance, to determine the effects of combined antioxidants, the effective concentration (EC_50_) values of each antioxidant gained from radical scavenging study (*e.g.*, DPPH) have to be inserted into the software in order to generate the CI value. The mathematical equation for CI is as follows:5
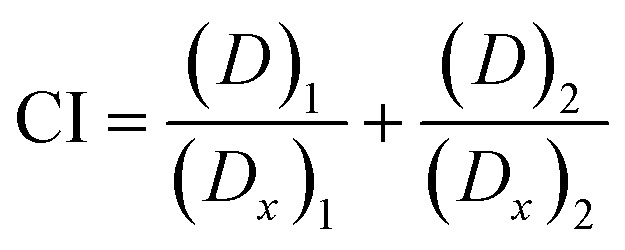
where (*D*_*x*_)_1_ and (*D*_*x*_)_2_ are the EC_50_ values of compound 1 and 2, respectively as a single agent, while (*D*)_1_ and (*D*)_2_ are the EC_50_ values of compound 1 and 2 in combination treatments.

### Efficiency of thymoquinone and/or l-ascorbic acid encapsulated in CNPs and PCNPs as radical scavengers in a human lung normal fibroblast (MRC-5) cell line by the reactive oxygen species (ROS) assay

#### Fluorescence microscope

About 100 000 cells were seeded overnight into each well of a 12-well plate containing 1 mL of complete grow media, CGM. On the following day, the cells were washed with cold PBS. Then, 500 μL of H_2_O_2_ of various concentrations (10 mM, 5 mM and 2.5 mM) was loaded into each well containing 500 μL of new CGM and it was incubated for 30 min at 37 °C, with 5% CO_2_. After the incubation, the cells were washed with cold PBS. Next, 900 μL of CGM (phenol red free) and 100 μL of CNP or PCNP samples were then loaded to provide antioxidant treatment for the cells. The treatment timepoints were set to 1 h, 24 h and 48 h. After reaching the timepoint, the CGM was removed and the cells were washed twice with cold PBS. Then, 10 μL of 5 μM CM_2_HDCF-DA ROS probe was loaded into the cells containing 990 μL of RPMI without serum. The plate was then incubated for 30 min at 37 °C, with 5% CO_2_. Cells were then washed twice with cold PBS and 1 mL of PBS was replaced. The plate was observed using a fluorescence microscope, with a fluorescein-5-isothiocyanate (FITC) option set. The assay and analysis were done in triplicate (*N* = 3), with three different synthesis batches. Throughout this experiment, phenol red–free RPMI media, supplemented with 10% FBS and 1% antibiotics, were used to reduce the interference with the fluorescence of CM-H_2_DCFDA.^38^

#### Flow cytometer

For flow cytometer analysis, the same preparation was made using a fluorescence microscope as stated above, but the volume was doubled as a 6-well plate was used and few additional steps were taken. The resulting cells were harvested using 250 μL trypsin (phenol red-free) and centrifuged at 1000 rpm for 5 min. Then, the cells were washed and resuspended in PBS in a 1.5 mL microcentrifuge tube. The intensity of the formed 2′7′-dichlorofluorescein (DCF) as a result of carboxy-DCFDA hydrolysis was analyzed using a NovoCyte flow cytometer and the NovoExpress software at excitation and emission wavelengths of 488 nm and 525 nm, respectively (NovoCyte, ACEA Bioscience, USA). An equivalent number of unstained cells were used as a blank. The assay and analysis were done in triplicate (*N* = 3), with three different synthesis batches.

### Statistical analysis

All results are expressed as mean ± standard error.

## Results and discussion

### Release of thymoquinone and l-ascorbic acid from CNP and PCNP carriers

**Fig. 4:**
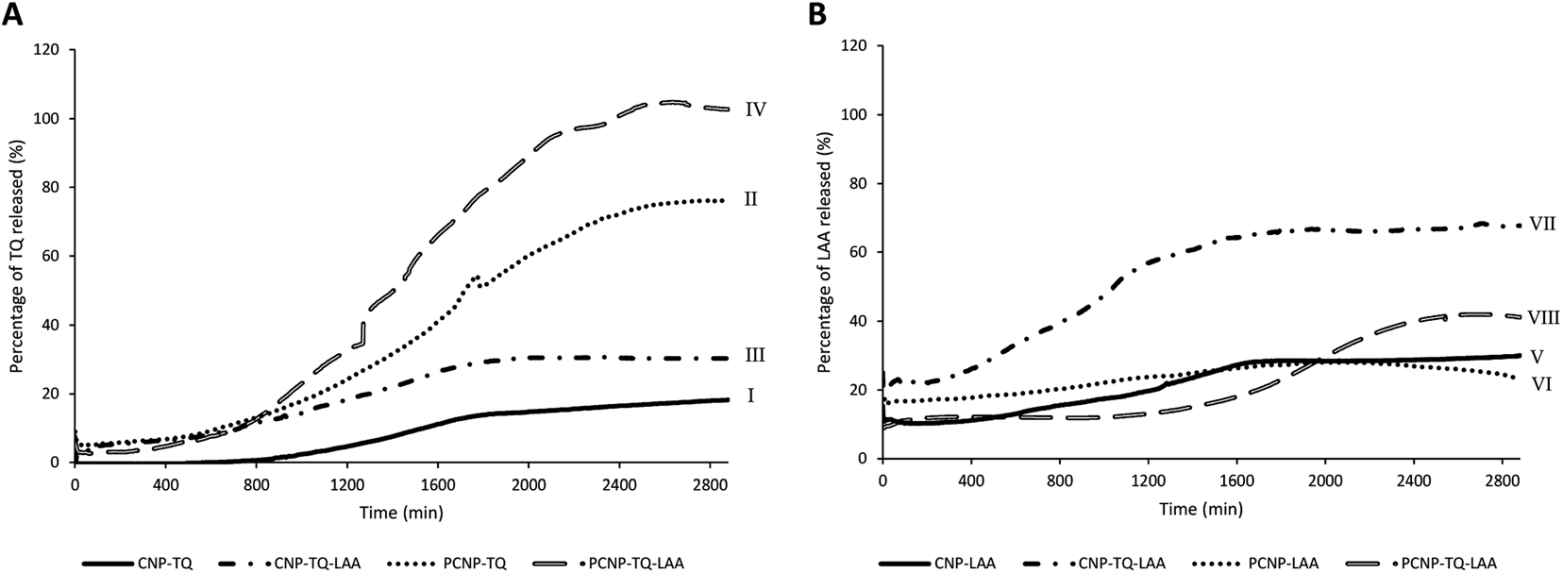
Release profiles of (A) TQ and (B) LAA from each sample, before and after CS modification with palmitic acid.

Release study was conducted to quantify how much of the encapsulated compound was released from the nanocarrier. The relationship between the encapsulated percentage of antioxidants based on previous reported studies^8,12^ on the released concentrations is shown in Table 1. The release profiles of TQ and LAA from CNP and PCNP carriers are shown in Fig. 4. Most of them had an initial burst release for the first 400 min, followed by slower and controlled release over a period of 48 h. A study reported on a similar pattern of CGA release from CNP.^39^ The initial burst release from chitosan-based NPs may be assigned to either adsorbed compounds on the surface of the NP or swelling of the polymer, which then created pores for the diffusion of compounds close to the NP surface (inside the polymer matrix).^39–42^ Additionally, hydrophobic TQ had a lower burst release effect in comparison to LAA because it had stronger affinity with the CNP or PCNP polymers due to lower water solubility,^43^ while the slow release was attributed to the NP matrix degradation or erosion and the encapsulated drug diffusion through the NP matrix.^44^ The differences in each antioxidant's release profile were influenced by changing the loaded antioxidant(s) and carrier type.

**Table 1:** Release percentages and concentrations of encapsulated TQ and LAA from CNP and PCNP samples. The encapsulated concentrations were acquired from previous published data^8,12^

Sample	Release of	Encapsulated	Released		
		Concentration (μM)	Concentration (μM)	Percentage ± SD (%)	Total time (h)
I	TQ from CNP-TQ	103.0	18.6	18.1 ± 7.2	46.8
II	TQ from PCNP-TQ	62.0	46.8	75.5 ± 1.0	43.9
III	TQ from CNP-TQ-LAA	53.4	16.3	30.5 ± 5.3	33.4
IV	TQ from PCNP-TQ-LAA	97.3	102.0	104.8 ± 8.0	43.9
V	LAA from CNP-LAA	110.9	31.4	28.3 ± 2.9	28.7
VI	LAA from PCNP-LAA	116.8	32.7	28.0 ± 4.2	33.7
VII	LAA from CNP-TQ-LAA	36.5	24.5	67.1 ± 5.1	30.1
VIII	LAA from PCNP-TQ-LAA	143.9	60.3	41.9 ± 0.0	43.7

In term of antioxidant changes, both TQ and LAA showed improved release percentages when the system was changed from single loaded to dual loaded, regardless of the type of carrier (CNPs or PCNPs). For instance, 12.4% more TQ was released from the dual-loaded CNP-TQ-LAA (sample III) than single-loaded CNP-TQ (sample I). The same observation was seen in PCNP carrier, where about 29.3% more TQ was released from dual-loaded PCNP-TQ-LAA (sample IV) than single-loaded PCNP-TQ (sample II). For LAA, the same trends were also detected when a dual-loaded system was used. Hence, the dual loaded system had increased the release efficiencies of both encapsulated antioxidants. This trend indicates that when TQ and LAA were encapsulated together in one carrier, they were released more efficiently, indirectly contributing to the increase in bioavailability. This could be supported by a study which discusses on higher releases of diclofenac sodium (DS) and dexamethasone (DX) when co-encapsulated together in biocompatible and biodegradable polymer PLGA NPs as compared to individually loaded DS or DX due to the synergistic therapeutic effects.^45^

In terms of the type of carrier selected, TQ released was significantly improved with the usage of modified PCNP carriers. Sample I and II were solely encapsulated with TQ, but with different carriers. By using the PCNP carrier, sample II had successfully increased the release percentage to about 57.5%. Hence, the modification of CS with palmitic acid to produce PCNPs had enhanced both encapsulation and release efficiencies. This situation is closely related to the outcomes that revealed the increased EE and release efficacies when changing the carrier from CNPs to PCNPs for the encapsulation of a hydrophobic lung cancer drug, silibinin.^46^ Even a higher percentage of about 70.0% more TQ were released by the dual-loaded system of the PCNP carrier (sample IV) than the CNP carrier (sample III) of the same co-loaded antioxidants, TQ and LAA. However, different observations were seen in LAA release profiles. The release of single-loaded LAA from both CNP (sample V) and PCNP (sample VI) carriers did not show significant differences. While LAA released from the dual-loaded CNP carrier (sample VII) had higher efficiencies than that of LAA released from the dual-loaded PCNP carrier (sample VIII). It could be contributed by the fact that the concentration of encapsulated LAA in the CNP-TQ-LAA was too little, 36.5 μM and way lower than the LAA encapsulated in the PCNP-TQ-LAA, 143.9 μM. Therefore, to maximize the release of LAA from the dual-loaded CNP carrier was easier as lesser amounts of compounds were entrapped. When a lower concentration of LAA was encapsulated in the CNP, lesser effort was needed to get all the compound released, which justifies higher release percentage than LAA released from PCNP carrier.^8^

### Diphenylpicrylhydrazyl (DPPH) radical scavenging using thymoquinone and/or l-ascorbic acid in CNPs *vs.* PCNPs

**Fig. 5:**
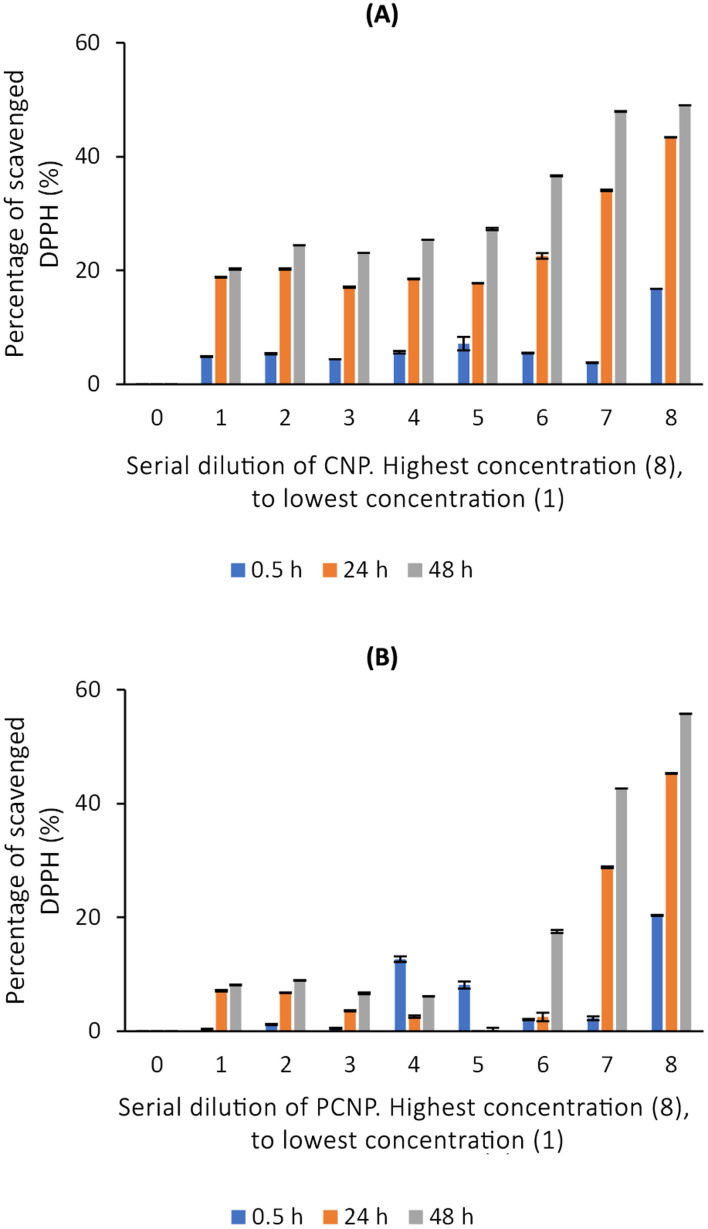
Percentage of DPPH scavenged by (A) CNPs and (B) PCNPs.

DPPH scavenging activity was first tested with the carriers itself, CNPs and PCNPs, as shown in Fig. 5. Concentration 1 to 4 had no trend because the carrier was too diluted. However, a stable increment was observed at concentration 5 onwards. Concentration 8 had the lowest standard error mean because the carrier concentration was sufficient to scavenge the radicals and can be detected using a microplate reader. CNPs did not only work as compound(s) carrier, but also as an antioxidant agent. This could be supported by few studies that reported on exhibition of CS antioxidant activity in scavenging radicals due to the presence of hydrogen atoms at C-2 (NH_2_), C-3 (OH), C-6 (OH) and protonated amino groups.^13,47,48^ Therefore, the CS is indeed an effective polymer to make antioxidant nanocarriers, as it augmented the free radical scavenging activities. Overall, PCNPs scavenged lesser DPPH than CNPs because the structure was more complex (with palmitoyl groups conjugated to the amines of CS), which reduced the hydrogen sources.^8^

The scavenging activity was then further observed with the usage of TQ compound as depicted in Fig. 7. Fig. 7A shows the bare TQ scavenging activity; at 0.5 h, no trend was observed in comparison to 24 h and 48 h, where increased concentrations resulted in higher DPPH scavenging. For 0.5 h, the highest percentage of scavenged DPPH was 11.07% at the highest TQ concentration of 78 080 μM. The differences observed were not obvious as it was too early for TQ to react with the radicals. However, at 24 h, the percentage of DPPH scavenged increased to at least 4 times from the 0.5 h performance. It tells that after 24 h, most TQ were available and ready to scavenge the radicals. Nonetheless, at 48 h, not much increment in terms of DPPH scavenged was seen as compared to 24 h because the presented TQ was maximal.

**Fig. 6:**
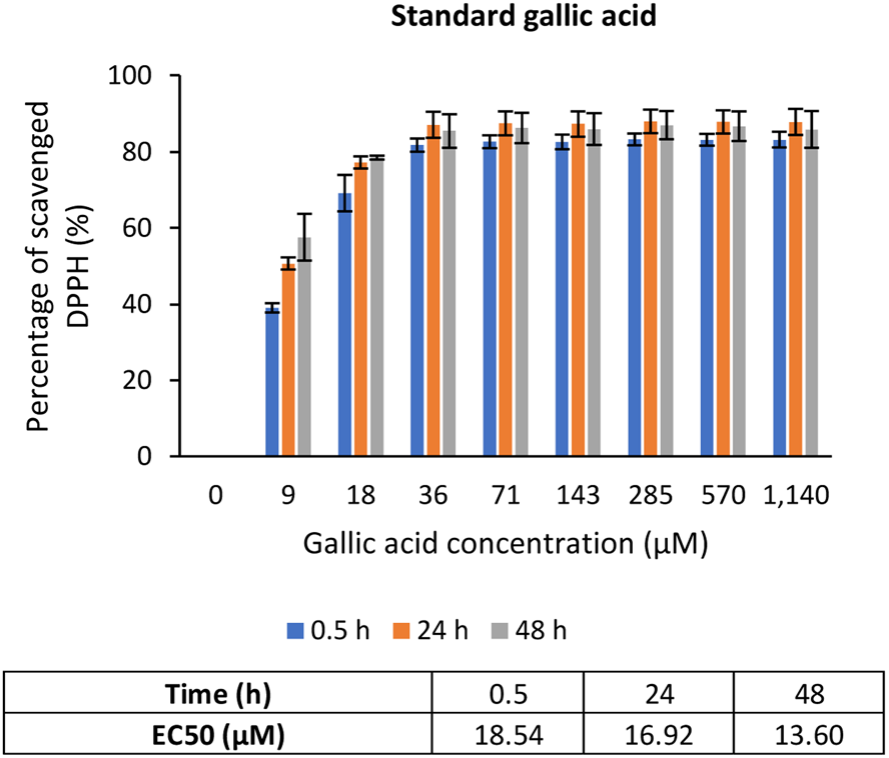
Percentage of DPPH scavenged by positive control, gallic acid.

Gallic acid was used as the standard in this assay since it possesses stable reactivity towards DPPH radicals.^49^ As shown in Fig. 6, the percentage of DPPH scavenged exhibited an upward trend from a concentration of 0 to 36 μM, after which it became a plateau, indicating equilibrium. This justifies that a low concentration of gallic acid was sufficient to reduce the radicals. Additionally, the EC_50_ value of gallic acid reduced over time, from 18.54 μM at 0.5 h to 16.92 μM at 24 h to 13.60 μM at 48 h, which means with time, less gallic acid was needed to scavenge DPPH radicals. According to the literature, the EC_50_ value of gallic acid was reported to be in the range of 5.73 μM to 29.5 μM.^49–51^ Undoubtedly, gallic acid is a potent antioxidant that can efficiently scavenge free radicals in the presence of its gallate group.^50,51^

**Fig. 7:**
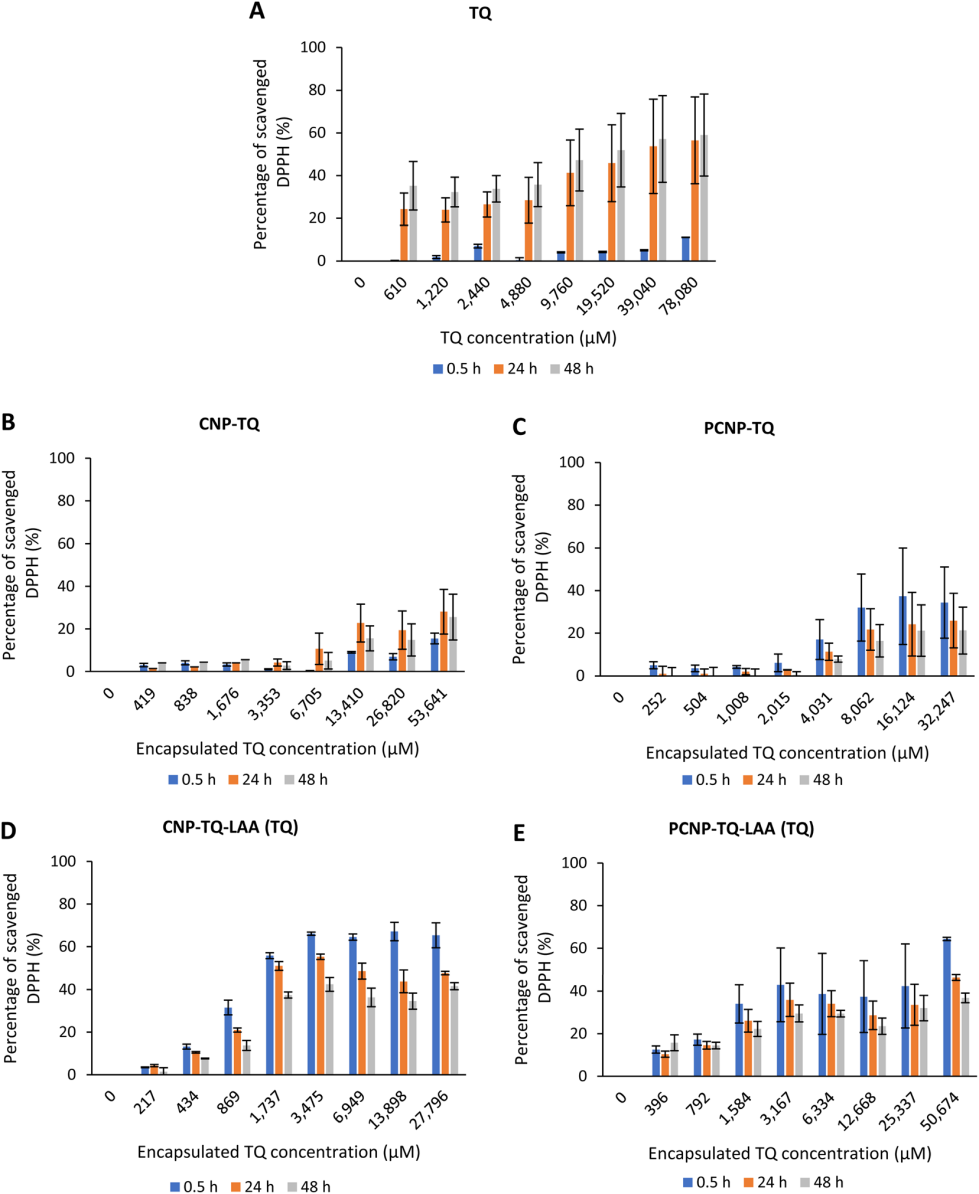
Percentage of DPPH scavenged by (A) TQ without any carrier. While, (B–E) are the percentage of DPPH scavenged by TQ from CNP-TQ, PCNP-TQ, CNP-TQ-LAA and PCNP-TQ-LAA, respectively. For (B–E), the encapsulated TQ concentration range was used as the *x*-axis, instead of loaded TQ concentration to measure the ability of encapsulated TQ only in scavenging DPPH radicals. Then, the percentage of TQ released was taken into account in calculating the final EC_50_ value at three different time points, 0.5, 24 and 48 h, as recorded in Table 2.

However, Fig. 7B–E show the scavenging capacities of TQ encapsulated in nanocarriers. The values of scavenged DPPH represent the performance of TQ solely, after eliminating the carrier's (CNP or PCNP) percentage of scavenged DPPH, as depicted in Fig. 5. The original performance of encapsulated TQ in carrier (without deducting carrier's percentage of scavenged DPPH) is shown in Appendix I (Fig. 15). The trend in the original data aligns with the trend of bare TQ in Fig. 7A, which means the DPPH scavenged increased over concentrations and time. After the scavenging activity of the carrier was deducted, the TQ performances (except CNP-TQ, Fig. 7B) increased over concentrations but decreased over time because with time, the carrier was able to scavenge higher DPPH radicals; hence, a higher percentage was deducted. On a side note, at 0.5 h, the scavenging activity was the highest as compared to 24 h and 48 h. This was influenced by the burst release effect of the TQ, as discussed in Release of thymoquinone and l-ascorbic acid from CNP and PCNP carrier section, Fig. 4A.

**Fig. 8:**
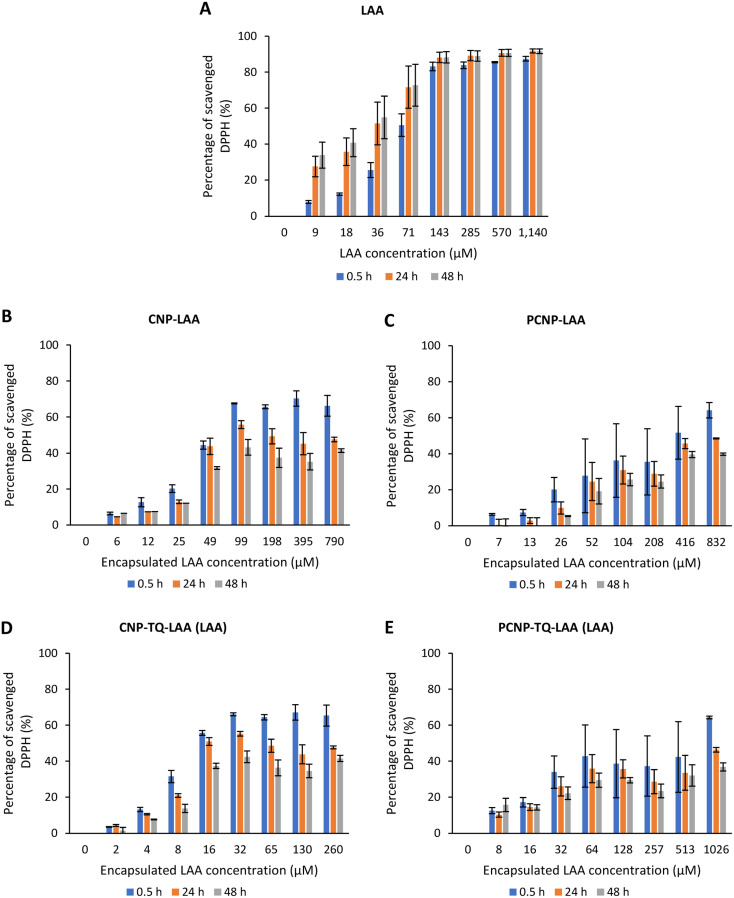
Percentage of DPPH scavenged by (A) LAA without any carrier. While, (B–E) are the percentages of DPPH scavenged by LAA from CNP-LAA, PCNP-LAA, CNP-TQ-LAA and PCNP-TQ-LAA, respectively. For (B–E), the encapsulated LAA concentration range was used as the *x*-axis, instead of the loaded LAA concentration to measure the ability of encapsulated LAA only in scavenging DPPH radicals. Then, the percentage of LAA released was taken into account in calculating the EC_50_ value at three different time points, 0.5, 24 and 48 h, as recorded in Table 2.

The DPPH scavenging activity by LAA is shown in Fig. 8. Fig. 8A depicts the bare LAA scavenging activity without any nanocarrier usage. Increased concentration of LAA had steadily increased the percentage of scavenging the DPPH radicals. The scavenging of DPPH radicals then started to plateau at 143 μM concentration. Furthermore, at all time points, LAA had a higher DPPH scavenging ability than that of TQ. It could be supported by a finding that LAA had 79 times higher antioxidant potential than TQ.^52^

The capacities of encapsulated LAA in CNPs or PCNPs are shown in Fig. 8B–E. The values of scavenged DPPH represent the performance of LAA solely, after eliminating the carrier's (CNPs or PCNPs) percentage of scavenged DPPH, as depicted in Fig. 5. The original performance of encapsulated LAA in carrier (without deducting carrier's percentage of scavenged DPPH) is shown in Appendix II (Fig. 16). The trend in the original data is the same as the trend in Fig. 8A, which means the DPPH scavenged increased over concentrations and time. After the scavenging activity of the carrier was deducted, the LAA performances increased over concentrations but decreased over time because with time, the carrier was able to scavenge higher DPPH radicals; hence, a higher percentage was deducted. Furthermore, at 0.5 h, the scavenging activity was the highest as compared to 24 h and 48 h. This was influenced by the burst release effect of the LAA, as discussed in Fig. 4B. However, the burst release effect (blue bars) at 0.5 h was reduced in the dual-loaded system, when the PCNP (Fig. 8E) carrier was used instead of the CNP (Fig. 8D). This explains that the modification of CS with palmitic acid effectively reduced the initial burst release of the encapsulated compounds by prolonging the retention of compounds.^8,53^ This was also caused by enhanced controlled release property as correlated with the release study data shown in Fig. 4.

Based on the plotted percentage of scavenged DPPH *vs.* encapsulated compound concentration graphs in Fig. 7 and 8, EC_50_ were then calculated as described in eqn (4) in the Reactivity of diphenylpicrylhydrazyl (DPPH) radical towards thymoquinone and/or l-ascorbic acid encapsulated in CNP and PCNP by DPPH assay section. The release percentages of TQ and LAA from CNP and PCNP systems are shown in Table 1. The EC_50_ values are presented in Table 2 to see the differences when compounds were delivered with and without nanocarrier over two days. At 24 h, the EC_50_ value of TQ in dual-loaded PCNPs was 2457.69 μM, which is 3 times lower than the EC_50_ value of TQ in single-loaded PCNPs, 8151.19 μM and 6 times lower than EC_50_ of bare TQ, 15 476.19 μM. In addition, at 24 h, the EC_50_ value of LAA delivered using dual-loaded CNPs was 12.47 μM, which was almost 2 times lower than the EC_50_ value of LAA in single-loaded CNPs, 18.31 μM and almost 5 times lower than EC_50_ of bare LAA, 55.70 μM.

**Table 2:** Effective concentration (EC_50_) of TQ and LAA in scavenging DPPH radicals when delivered without and with nanocarrier (CNPs or PCNPs) over 48 h. A lower EC_50_ value resembles a higher scavenging efficiency, which requires a lower concentration of compound to scavenge the same amount of DPPH radicals

Compound	EC_50_ (μM)								
	0.5 h			24 h			48 h		
	Bare	Single	Dual	Bare	Single	Dual	Bare	Single	Dual
TQ	208 333.33	CNP		15 476.19	CNP		9346.61	CNP	
		365.20	41.48		1864.32	492.75		11 033.89	965.74
		PCNP			PCNP			PCNP	
		493.74	57.22		8151.19	2457.69		36 769.45	8886.63
LAA	81.46	CNP		55.70	CNP		42.47	CNP	
		7.77	4.43		18.31	12.47		28.17	20.16
		PCNP			PCNP			PCNP	
		25.50	3.86		56.66	11.22		106.25	33.62

Almost all EC_50_ reduced when the compound was delivered using single-loaded NPs and even lower when using dual-loaded NPs, in comparison to bare delivery, regardless of the type of nanocarrier (CNP or PCNP). Hence, the CS-based nanocarrier containing dual compounds worked most efficiently in scavenging DPPH radicals than single-loaded and bare compounds. Likewise, an increase in DPPH scavenging activity was seen as a result of using NPs to contain quercetin as compared to the bare delivery of quercetin^54^. In another study, DPPH scavenging activity increased when silver nanoparticles (AgNPs) were synthesized using a *P. farcta* fruit extract in comparison to the DPPH scavenging activity using the *P. farcta* fruit only.^55^ Additionally, this may be due to the positive combination effects of both compounds or known as synergy. It can be determined through the CI study in the next section. However, at 24 h and 48 h, LAA delivered using single-loaded PCNPs had higher EC_50_ (56.66 μM and 106.25 μM, respectively) than the bare LAA (55.70 μM and 42.47 μM, respectively). This is due to the fairly low percentage of LAA released from the PCNP, as shown in release data Fig. 3. With lower ability to release LAA from the carrier, lesser DPPH were scavenged.

Other than that, higher EC_50_ was recorded when the carrier was changed from CNPs to PCNPs. For instance, at 24 h, the EC_50_ value of dual-loaded TQ delivered by PCNPs, 2457.69 μM, was 5 times higher than the EC_50_ value of dual-loaded TQ delivered by CNPs, 492.75 μM. It can be influenced by the 30.0% higher release by the PCNP that needs to be taken into account in calculating the final EC_50_ value. The release percentage had to be included in the calculation since the compound had to be released first, before it could scavenge the radicals. Moreover, the conjugation of palmitic acid on CS to make PCNPs had increased the retention of compounds, lengthening the period of compound staying in the carrier for more steady controlled release.^53^ On a side note, all systems showed an increase in EC_50_ over time. For instance, for PCNP-TQ (Fig. 6C), the value of EC_50_ increased from 0 μM at 0.5 h to 8903.34 μM at 24 h to 23 710.44 μM at 48 h because with time, lesser TQ left, so in order to reach 50% of radical scavenging, more TQ was needed.

The reduction reaction of the DPPH radical took place when it received hydrogen from the antioxidant(s), resulting in the formation of DPPH-H (non-radical) and changed its color from purple to yellow.^17,19,56^ Based on the EC_50_ values, the DPPH scavenging activity shown by LAA was higher than TQ because TQ has a lower capacity of work as antioxidant hydrogen or electron donors.^52^ LAA is reported to scavenge DPPH radicals in MeOH by a hydrogen transfer reaction to form DPPH-H.^18^ Some studies reported that the EC_50_ value of LAA was around 20 μM for the DPPH assay.^52,57^ TQ, however, scavenged DPPH radicals by hydrogen or electron donation.^52^ Since this study involved DPPH scavenging over 48 h, DMSO was selected as the most suitable solvent for the DPPH powder as compared to methanol because it is less volatile and has a lower evaporation rate at room temperature.^58,59^

### Increased efficacy of DPPH scavenged by the combination of TQ and LAA in PCNP through synergistic effects

Combining two compounds, TQ and LAA, in a single carrier (CNP or PCNP) led to the inquiry on its interaction effects. Mathematical calculation known as CI is commonly used to determine interactions, as shown in eqn (5). The combined effects can be determined as either synergistic (CI < 1), antagonistic (CI > 1) or additive (CI = 1).^20^ In this study, the EC_50_ values derived from the DPPH assay were used for the CI calculation, and the results are shown in Table 3 (the example of CI calculation is shown in Appendix III). All CI values were less than 1, which indicates that TQ and LAA showed synergistic effects in both CNP and PCNP dual-loaded systems, at all time points. The synergy was evident as EC_50_ (for instance at 24 h) of both TQ and LAA delivered by dual-loaded CNPs (492.75 μM and 12.47 μM, respectively) was lower than EC_50_ of TQ and LAA delivered by single-loaded CNPs (1864.32 μM and 18.31 μM, respectively). The same scenario was observed in the PCNP carrier, where the EC_50_ values of TQ and LAA delivered by the dual-loaded system (2457.69 μM and 11.22 μM, respectively) were lower than EC_50_ of TQ and LAA delivered by the single-loaded system (8151.19 μM and 56.66 μM, respectively). The same observations were identified in the EC_50_ trends of synergistic effects of co-delivering docetaxel and salinomycin in a NP consisting of poly lactide-co-glycolide/D-alpha-tocopherol polyethylene glycol for breast cancer treatment.^37^

**Table 3:** Classical isobologram (CI) analysis of DPPH radicals scavenged by a combination of TQ and LAA by CNPs and PCNPs. The combined effects can be classified as synergistic (CI < 1), antagonistic (CI > 1) or additive (CI = 1)

	Time (h)	0.5	24	48
CNP	EC50 of TQ in dual loaded CNP-TQ-LAA (μM)	41.48	492.75	965.74
	EC50 of TQ in single loaded CNP-TQ (μM)	365.20	1864.32	11 033.89
	EC50 of LAA in dual loaded CNP-TQ-LAA (μM)	4.43	12.47	20.16
	EC50 of LAA in single loaded CNP-LAA (μM)	7.77	18.31	28.17
	CI value of TQ and LAA in dual loaded CNP-TQ-LAA	0.68	0.95	0.80

PCNP	EC50 of TQ in dual loaded PCNP-TQ-LAA (μM)	57.22	2457.69	8886.63
	EC50 of TQ in single loaded PCNP-TQ (μM)	493.74	8151.19	36 769.45
	EC50 of LAA in dual loaded PCNP-TQ-LAA (μM)	3.86	11.22	33.62
	EC50 of LAA in single loaded PCNP-LAA (μM)	25.50	56.66	106.25
	CI value of TQ and LAA in dual loaded PCNP-TQ-LAA	0.15	0.50	0.56

The CI values in the CNP system increased from 0.68 at 0.5 h to 0.95 at 24 h and decreased to 0.80 at 48 h. It could be said that the scavenging effects of TQ and LAA in the CNP system were unpredictable and influenced by the release kinetics. However, the trend was not parallel in the PCNP system. The values of CI increased with time, from 0.15 at 0.5 h to 0.50 at 24 h to 0.56 at 48 h. This tells that the synergistic effects reduced when lesser compounds were available at longer time points, since most of them had been used up. If the reaction duration was to be lengthened, the scavenging effects of TQ and LAA in the PCNP system may change to addition (CI = 1), instead of synergism. On a side note, changing the carrier from CNPs to PCNPs led to a reduction in CI values. For instance, at 24 h, the CI value reduced to almost 50% from 0.95 to 0.50. Thus, it denotes that the interaction between the antioxidant compounds was more synergistic in the modified carrier, PCNP than the CNP. This proves that the conjugation of palmitic acid on CS contributed to the production of more effective dual-loaded compounds encapsulated in a nanocarrier for DPPH radical scavenging.

Overall, the CI values of combined TQ and LAA by PCNP carriers were lower than those by CNP carriers. Thus, the modification of CS with palmitic acid had contributed to the production of more synergistic interactions between the encapsulated antioxidant compounds. This is supported by the fact that the closer the CI value to 1, the lesser its synergistic effects. Additionally, an increase in the synergistic effects was reported when ascorbic acid was delivered in combination with glucose and encapsulated in CS nanoformulations to scavenge DPPH radicals.^60^ In another study, combining doxorubicin with formaldehyde releasing prodrug, AN-250, encapsulated in CNPs resulted in enhanced delivery through synergistic relationship for cancer treatment.^53^ These outcomes concluded that an increase in drug delivery efficiencies was influenced by combining drugs using chitosan-based NPs as compared to single delivery or bare delivery. In this study, enhanced scavenging abilities of combined TQ and LAA were caused by the synergistic effects when delivered by a NP system.

### Thymoquinone and/or l-ascorbic acid in PCNPs as radical scavengers in a normal human lung fibroblast cell line, MRC-5

**Fig. 9:**
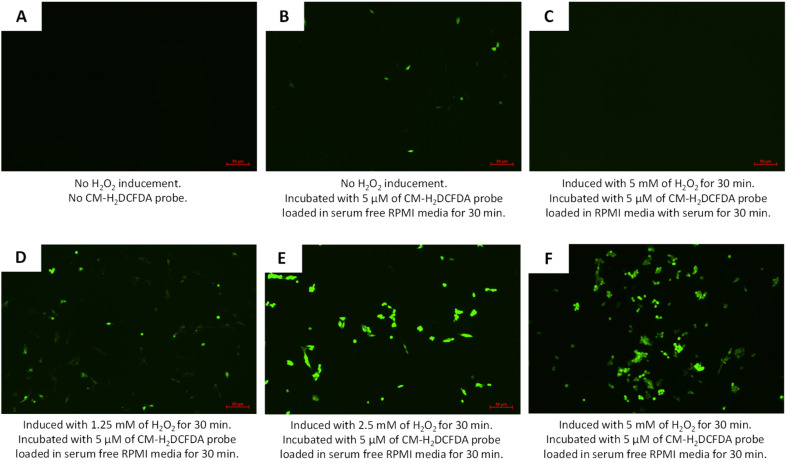
Green dichlorodihydrofluorescein (DCF) expression as a result of ROS presence in MRC-5 when (A) no CM-H_2_DCFDA probe loaded, (B) no H_2_O_2_ induced but incubated for 30 min with 5 μM of CM-H_2_DCFDA probe loaded in serum-free RPMI media, (C) induced with 5 mM H_2_O_2_ and then incubated for 30 min with 5 μM of CM-H_2_DCFDA probe loaded in serum supplemented RPMI media, (D) induced with 1.25 mM H_2_O_2_ and then incubated for 30 min with 5 μM of CM-H_2_DCFDA probe loaded in serum-free RPMI media, (E) induced with 2.5 mM H_2_O_2_ and then incubated for 30 min with 5 μM of CM-H_2_DCFDA probe loaded in serum-free RPMI media, and (F) induced with 5 mM H_2_O_2_ and then incubated for 30 min with 5 μM of CM-H_2_DCFDA probe loaded in serum-free RPMI media.

Enhanced synergy between TQ and LAA delivered using hydrophobically modified PCNPs in scavenging DPPH radicals was worthily further experimented by an *in vitro* method. The reactive oxygen species (ROS) assay using CM-H_2_DCFDA, an intracellular probe, was conducted to determine free radical scavenging activity by TQ and LAA within a human lung fibroblast cell line, MRC-5. A preliminary study on the functionality of the CM-H_2_DCFDA probe was qualitatively tested using a fluorescence microscope. The probe was first tested whether it could emit the fluorescence when incubated with or without serum-supplemented media. Fig. 9 shows the expression of the probe when set to different conditions. Fig. 9B shows that the oxidized cells emitted green DCF when the CM-H_2_DCFDA probe was loaded in serum-free RPMI media (even without H_2_O_2_ inducement). Meanwhile, in Fig. 9C, the MRC-5 cells induced with 5 mM H_2_O_2_ showed no fluorescein emission as the media used contained serum.

A higher concentration of ROS was needed to enable DCF fluorescence emergence when H_2_DCFDA was used with an RPMI media containing serum, as compared to using serum-free RPMI media. In addition, serum provided extracellular esterases that converted more H_2_DCFDA into H_2_DCF, which then eased the oxidation by the ROS and resulted in higher background fluorescence.^61^ Moreover, H_2_DCFDA is very sensitive to certain compounds such as bovine serum albumin, heme and serum.^62,63^ Thus, the usage of serum-free media during CM-H_2_DCFDA probe loading is essential for the least emergence of background fluorescence. On top of that, by inducing the cells with hydrogen peroxide, H_2_O_2_, emergence of fluorescein, DCF could be detected more clearly. The increase in H_2_O_2_ concentration led to an increase in the intensity of fluorescein, DCF, as shown in Fig. 9 D–F.

**Fig. 10:**
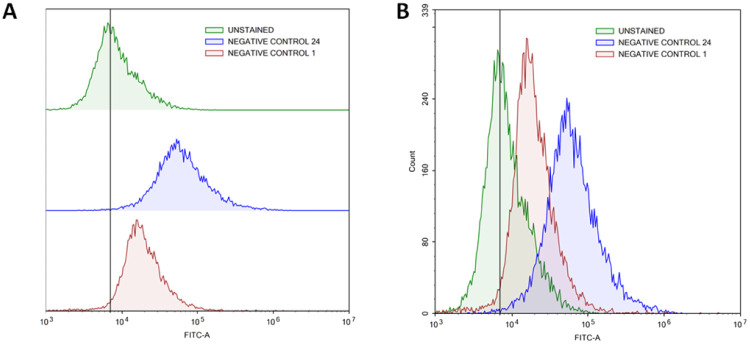
Increased ROS level in MRC-5 induced with H_2_O_2_ for 24 h. (A) represents the progression of ROS levels through the shift of peak (right shift means higher ROS presence), while (B) represents the number of cells at a particular FITC-A intensity. A higher FITC-A reading indicates more DCF or ROS presence. MRC-5 induced with H_2_O_2_ for 24 h had a greater FITC-A intensity than induced for 1 h, which resembles higher ROS presence.

The radical scavenging in MRC-5 by TQ and/or LAA encapsulated in PCNP was then further quantified using flow cytometry at two time points, 24 h and 48 h. Firstly, the H_2_O_2_ inducement duration was optimized to determine the most suitable time needed to get a significant level of radicals that could be detected by the flow cytometer. Fig. 10A shows that the peak of cells induced with H_2_O_2_ for 24 h (blue peak) shifted to the right more than the 1 h (red peak). It defines that the ROS level (depicted as FITC-A) increased with longer exposure of the H_2_O_2_. Instead, not much DCF was produced when the cells were induced with H_2_O_2_ for 1 h only. Hence, the 24 h of H_2_O_2_ inducement on MRC-5 was used in the following experiments.

**Fig. 11:**
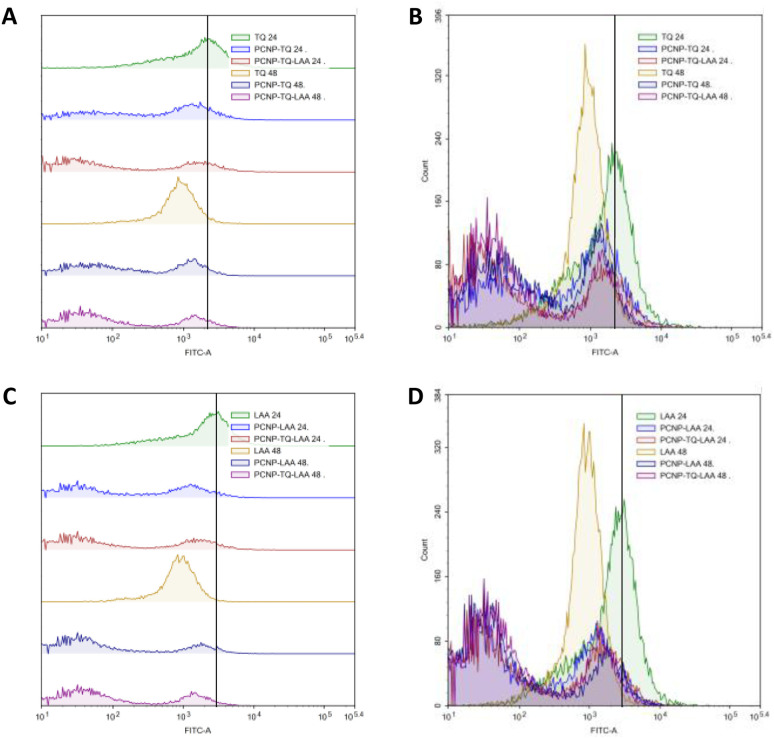
Effect of TQ and/or LAA treatments with or without usage of PCNP carriers for 24 h and 48 h on the ROS level in MRC-5. (A) and (B) are of the same samples (LAA scavenging activities), (C) and (D) are of the same samples (TQ scavenging activities). (A) and (C) represent the progression of the ROS level through the shift of peak (right shift means higher ROS presence), while (B) and (D) represent the number of cells at a particular FITC-A intensity. Higher FITC-A reading indicates higher DCF or ROS presence. MRC-5 treated with samples for 48 h had higher radical scavenging activities than 24 h due to the controlled release of compounds. The dual-loaded TQ and LAA in PCNPs (PCNP-TQ-LAA) at 48 h showed the lowest ROS level because of the synergistic effects of the combination.

**Fig. 12:**
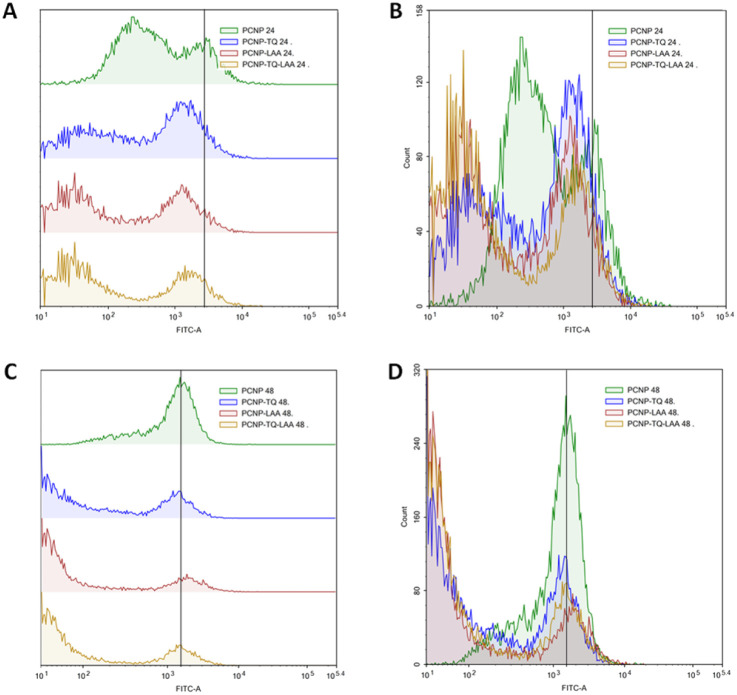
Scavenging of H_2_O_2_ by compound(s)-encapsulated PCNPs. (A) and (B) are of the same samples (24 h post treatment), (C) and (D) are of the same samples (48 h post treatment). (A) and (C) represent the progression of the ROS level through the shift of peak (right shift means higher ROS presence), while (B) and (D) represent the number of cells at a particular FITC-A intensity. A higher FITC-A reading indicates more DCF or ROS presence. Two peaks were detected from each treatment. MRC-5 treated for 48 h in C showed higher scavenging activities as the right peak areas were smaller than those treated for 24 h in A. It indicates that 48 h post treatment caused the ROS to be reduced more efficiently as more cells accumulated at the left peaks with lower FITC-A values.

**Fig. 13:**
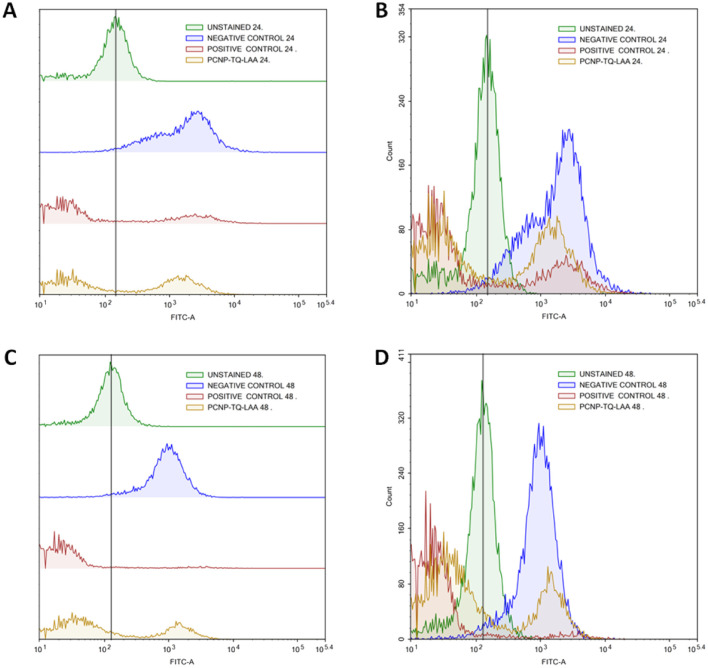
Scavenging of H_2_O_2_ by dual-loaded TQ and LAA in PCNPs. (A) and (B) are of the same samples (24 h post treatment), (C) and (D) are of the same samples (48 h post treatment). (A) and (C) represent the progression of ROS level through the shift of peak (right shift means higher ROS presence), while (B) and (D) represent the number of cells at a particular FITC-A intensity. Higher FITC-A reading indicates more DCF or ROS presence. MRC-5 treated with dual-loaded TQ and LAA in PCNP for 48 h in (C) showed higher scavenging activities as the right peak area was smaller than treated for 24 h in (A). It indicates that 48 h post treatment caused the ROS to be reduced more efficiently as more cells accumulated at the left peak with lower FITC-A values.

MRC-5 were induced with H_2_O_2_ for 24 h, before treatment with TQ and/or LAA delivered as the bare compound, single-loaded PCNP or dual-loaded PCNP. The ROS generated 24 h and 48 h post treatment were analyzed by Flow Cytometry and are presented in Fig. 11–13.

**Fig. 14:**
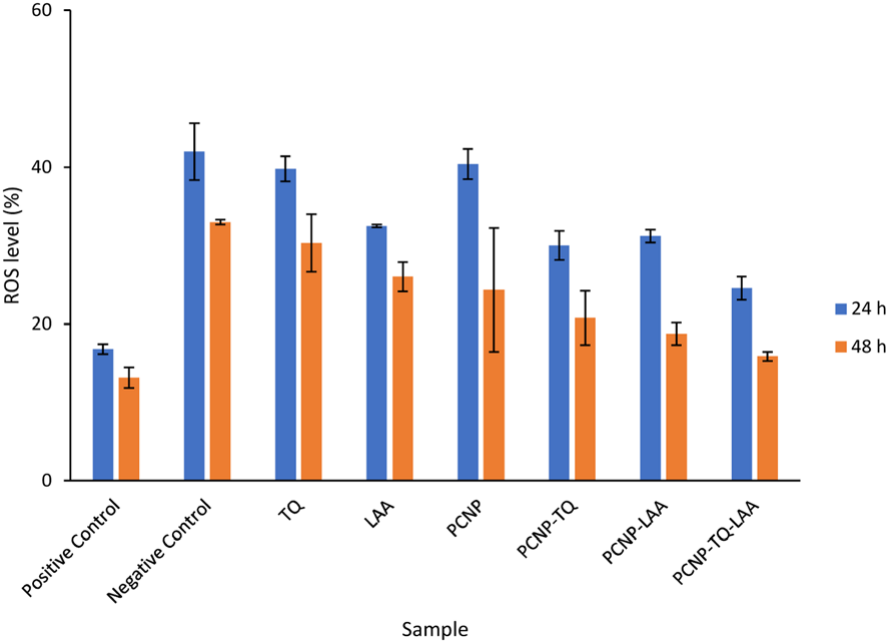
Percentage of ROS in MRC-5 after being induced with H_2_O_2_ for 24 h and treated with various treatments over 24 h and 48 h. The positive control, gallic acid, had the least ROS, while the negative control H_2_O_2_ had the most ROS post treatment. The ROS level dropped in all samples after treatment for 48 h in comparison to 24 h, which indicates higher scavenging activities. The percentages of ROS are from the DCF fluorescence histograms (Fig. 10–13) analyzed using the FlowJo analysis program. The percentage of ROS was quantified considering a threshold fluorescence level of 10^3^ (a.u), set on the basis of the background fluorescence in the unstained cell population. The ROS values in each sample are shown in Table 4.

The compiled ROS levels in MRC-5 24 h and 48 h post treatments of various samples are summarized in Fig. 14 and Table 4. The positive control, gallic acid, had the least ROS level, which indicates high efficiency in scavenging H_2_O_2_ radicals, since it is known for its potent antioxidative property. The negative control, H_2_O_2_, however showed the highest ROS level as MRC-5 were induced with H_2_O_2_, but not being treated with any antioxidant. Therefore, the oxidized cells containing free radicals were not reduced to stable form radicals. After 24 h, the ROS level in MRC-5 treated with just PCNPs (40.38%) had almost the same percentage as the negative control (41.98%), which scavenged only 1.60% of H_2_O_2_. However, after 48 h, the PCNP scavenged around 18.00% of H_2_O_2_. It means that more PCNPs were degraded at 48 h to scavenge H_2_O_2_.^64^

**Table 4:** Percentage of ROS in MRC-5 after being induced with H_2_O_2_ for 24 h and treated with various treatments over 24 h and 48 h. Positive control and negative control had the least and most ROS, respectively. By time, the ROS level dropped in all samples, which indicates more scavenging activities. The percentages of ROS are from the DCF fluorescence histograms (Fig. 10–12) analyzed using the FlowJo analysis program. The percentage of ROS was quantified considering a threshold fluorescence level of 10^3^ (a.u), set on the basis of the background fluorescence in the unstained cell population. SEM means standard error mean

Sample	ROS level ± SEM (%)	
	24 h post treatment	48 h post treatment
Positive control	16.80 ± 0.64	13.16 ± 1.32
Negative control	41.98 ± 3.61	32.98 ± 0.33
TQ	39.79 ± 1.60	30.29 ± 3.69
LAA	32.46 ± 0.19	26.00 ± 1.86
PCNP	40.38 ± 1.93	24.33 ± 7.88
PCNP-TQ	29.99 ± 1.85	20.74 ± 3.46
PCNP-LAA	31.20 ± 0.82	18.71 ± 1.43
PCNP-TQ-LAA	24.52 ± 1.47	15.88 ± 0.57

Both single-loaded compounds, PCNP-TQ and PCNP-LAA, had nearly similar ROS levels of 29.99% and 31.20%, respectively at 24 h. About 9.00% and 13.00% of ROS were further reduced by PCNP-TQ and PCNP-LAA, respectively 48 h post treatment, making the ROS level 20.74% and 18.71%, respectively. On top of this, the dual-loaded PCNP-TQ-LAA had 24.52% and 15.88% of ROS at 24 h and 48 h post treatment; which means around 9.00% more ROS decreased by time. The trend of ROS decrement over time in all compound(s)-encapsulated PCNP samples was caused by the controlled release system that retained the compound and released it slowly in longer periods of time. Additionally, since PCNPs showed a significant ROS decrement from 24 h (40.38%) to 48 h (24.33%) which might be due to the degradation, it hints that more encapsulated compounds were released by time.

On another note, bare TQ and LAA had higher ROS levels than the encapsulated ones in PCNPs. For instance, at 24 h, the ROS level in MRC-5 treated with bare TQ and LAA were 39.79% and 32.46%, respectively. However, when TQ and LAA were encapsulated as a single agent in PCNPs, the ROS level was reduced by 10.00% and 1.00%, respectively. The reduction was further enhanced when both TQ and LAA were delivered together in PCNPs, in which the ROS reading was 24.53%. That marks 15.00% and 8.00% decrement for TQ and LAA, respectively. The ROS were scavenged most efficiently by the dual-loaded system (PCNP-TQ-LAA) than the single-loaded system (PCNP-TQ, PCNP-LAA) because the combined compounds in PCNPs had higher release percentages (Release of thymoquinone and l-ascorbic acid from CNP and PCNP carrier section, Fig. 4). Moreover, the synergistic effects of combined TQ and LAA increased their capabilities to reduce the number of radicals in comparison to single loading in the PCNP and bare delivery. This was aligned with the findings from the DPPH assay in which the CI value shown by the combined TQ and LAA in PCNPs was < 1.

The increase in scavenging free radicals could be caused by many factors and one such factor is the enhanced bioavailability facilitated by a controlled release system. In this study, the bioavailability of TQ and LAA was enhanced by the use of PCNP carriers. It was proven when bare TQ and LAA had higher percentages of ROS as compared to when they were encapsulated. This is supported by a finding that TQ loaded in nanostructured lipid carriers had a 6-fold increase in bioavailability as compared to free TQ, which consequently provided better gastrointestinal protection.^65,66^ On top of that, PCNPs showed enhanced controlled release of dual-encapsulated TQ and LAA in comparison to single-encapsulated TQ or LAA. Hence, higher ROS were scavenged by the dual loaded system at both time points, 24 h and 48 h. The improvement was not solely contributed by the controlled release system, but by the synergy between TQ and LAA too.

## Conclusions

The antioxidant scavenging activities of TQ and LAA were measured by a DPPH assay. The compounds were administered as bare, single-loaded CNPs and PCNPs as well as dual-loaded CNPs and PCNPs. The DPPH scavenging activity in DMSO by bare LAA was higher than that of TQ. The efficacy experienced an increase when the compounds were administered using NPs, as they were protected. This elevated the retention and controllably released these substances over 48 h. The EC_50_ value of TQ and LAA from the dual-loaded PCNP and CNP in scavenging the synthetic DPPH radical was found to be lower than the EC_50_ value of single-loaded systems. It implies that the combination of TQ and LAA in the carrier reduced the concentration of antioxidants required to effectively scavenge the DPPH radicals. The dual-loaded TQ and LAA in both CNP and PCNP systems clearly exhibited synergistic effects when combined in one carrier as the CI values were less than one. However, the dual-loaded PCNP displayed higher synergistic effects based on the lower CI value scored as compared to the dual-loaded CNP. It defines that the modification of CS by palmitic acid had effectively enhanced the reduction of the free radical DPPH through higher activities of hydrogen transfer. Due to that, another *in vitro* antioxidative ROS assay was performed, called the CM-H_2_DCFDA assay to further determine TQ and LAA capabilities in scavenging radicals. In the ROS assay study, H_2_O_2_ was used as the free radicals to be scavenged by the reducing agent. Parallel outcomes were shown by those compounds, in which dual-loaded TQ and LAA in PCNPs had the highest scavenging activity. The ROS level was the lowest when TQ and LAA were administered in combination by the PCNP carrier, followed by single-loaded TQ and single-loaded LAA, bare TQ and bare LAA. Aside from the synergistic effects of the combination, the utilization of PCNPs as the carrier increased the retention duration, allowing the gradual release of the payload. This reduced the concentration of loaded compounds TQ and LAA needed to scavenge both DPPH and H_2_O_2_ radicals without reducing the efficacies. In conclusion, the controlled release property of the PCNP carrier and synergy between TQ and LAA compounds was very crucial to augment the overall antioxidative property of a system.

## Appendices

### Appendix I

**Fig. 15:**
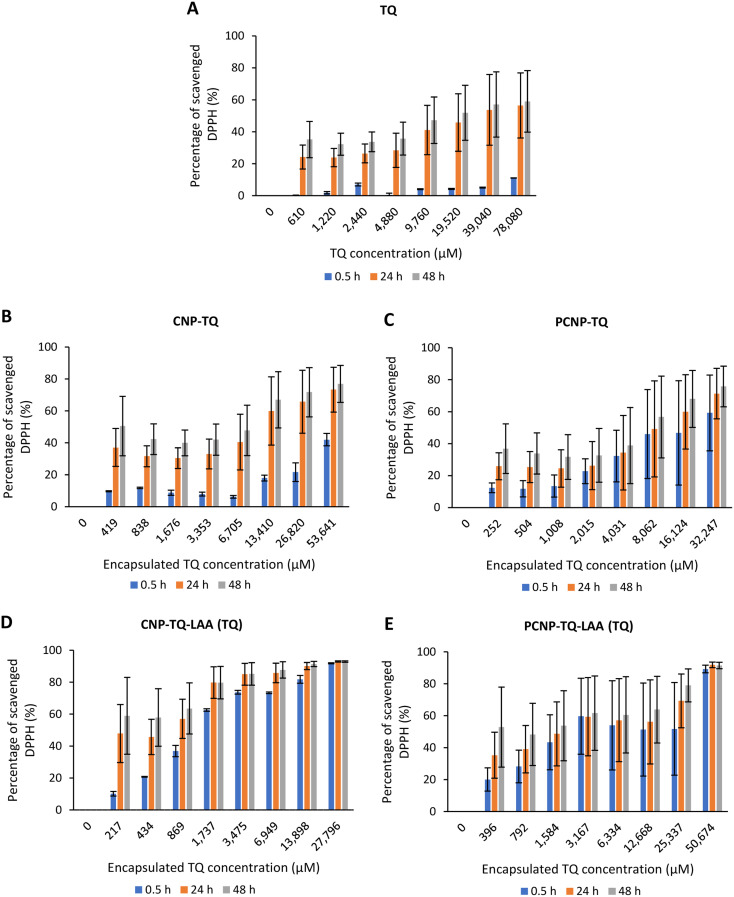
Original performance of encapsulated TQ in carrier (without deducting carrier's percentage of scavenged DPPH).

### Appendix II

**Fig. 16:**
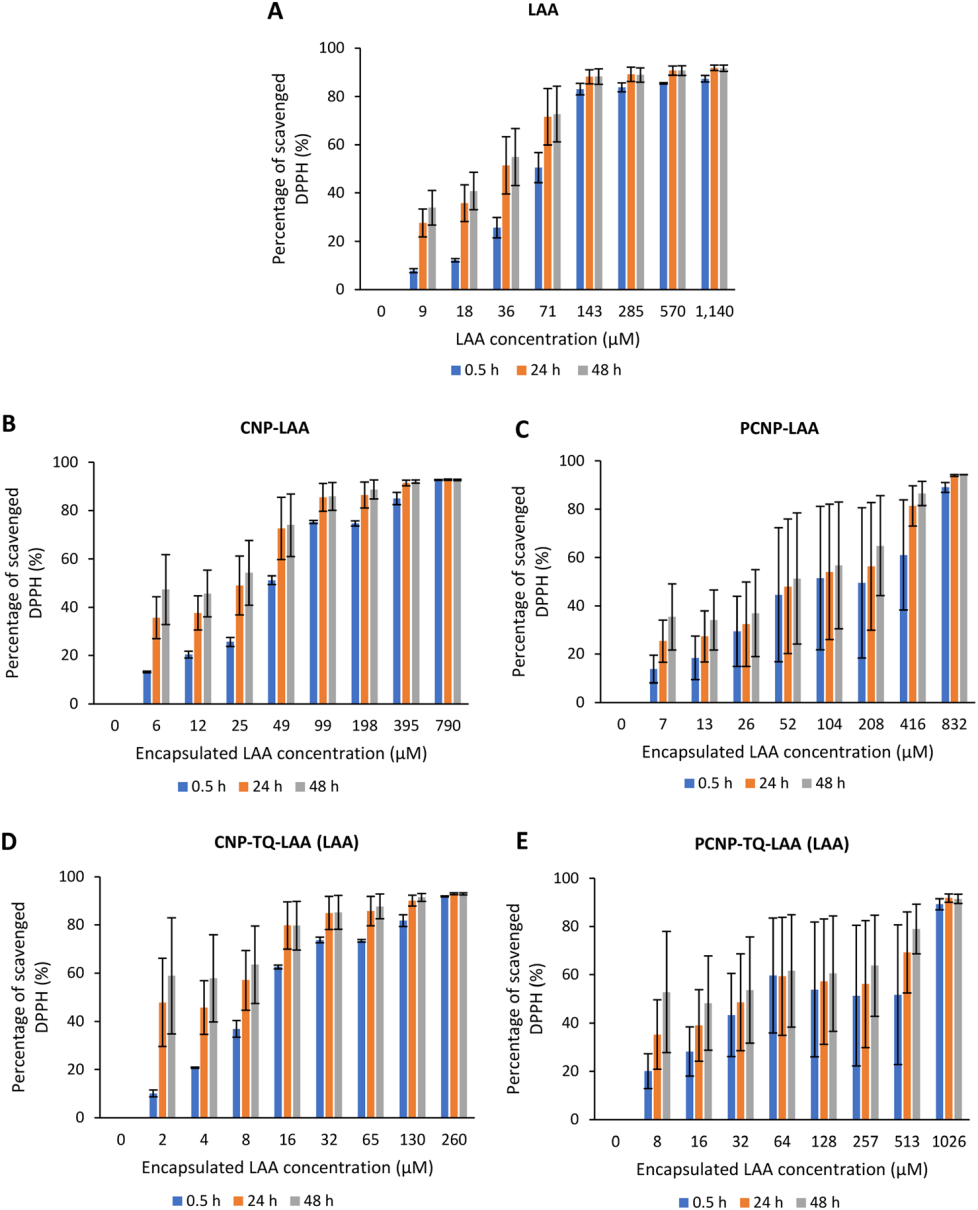
Original performance of encapsulated LAA in carrier (without deducting carrier's percentage of scavenged DPPH).

### Appendix III

Example of calculation for CI value of two compounds combined in a CNP carrier.

CI value of TQ and LAA in dual loaded CNP-TQ-LAA at 48 h.
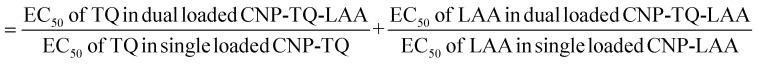

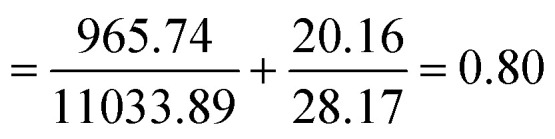


## Author contributions

Conceptualization and methodology: N. O., S. N. A. M. J. and M. J. M.; investigation and formal analysis: N. O.; software: N. O., R. A. B. M. J. and M. N. A.; supervision and resources: S. N. A. M. J. and M. J. M.; validation: S. N. A. M. J. M. J. M., R. A. B. M. J. and M. N. A.; writing – original draft: N. O.; writing – review & editing: S. N. A. M. J. All authors have read and agreed to the published version of the manuscript.

## Conflicts of interest

There are no conflicts to declare.

## Supplementary Material
